# Synergistic Effects of Nucleating Agents and Plasticizers on the Crystallization Behavior of Poly(lactic acid)

**DOI:** 10.3390/molecules20011579

**Published:** 2015-01-19

**Authors:** Xuetao Shi, Guangcheng Zhang, Thanh Vu Phuong, Andrea Lazzeri

**Affiliations:** 1 Department of Applied Chemistry, School of Science, Northwestern Polytechnical University, 127# Youyi XiLu, Xi’an 710072, China; E-Mail: zhangguc@nwpu.edu.cn; 2 Department of Chemical Engineering, Industrial Chemistry and Materials Science, University of Pisa, via Diotisalvi 2, Pisa 56126, Italy; E-Mail: thanhvu_phuong@yahoo.com

**Keywords:** poly(lactic acid), nanoparticles, particle-reinforced composites, synergism

## Abstract

The synergistic effect of nucleating agents and plasticizers on the thermal and mechanical performance of PLA nanocomposites was investigated with the objective of increasing the crystallinity and balancing the stiffness and toughness of PLA mechanical properties. Calcium carbonate, halloysite nanotubes, talc and LAK (sulfates) were compared with each other as heterogeneous nucleating agents. Both the DSC isothermal and non-isothermal studies indicated that talc and LAK were the more effective nucleating agents among the selected fillers. Poly(d-lactic acid) (PDLA) acted also as a nucleating agent due to the formation of the PLA stereocomplex. The half crystallization time was reduced by the addition of talc to about 2 min from 37.5 min of pure PLA by the isothermal crystallization study. The dynamic mechanical thermal study (DMTA) indicated that nanofillers acted as both reinforcement fillers and nucleating agents in relation to the higher storage modulus. The plasticized PLA studied by DMTA indicated a decreasing glass transition temperature with the increasing of the PEG content. The addition of nanofiller increased the Young’s modulus. PEG had the plasticization effect of increasing the break deformation, while sharply decreasing the stiffness and strength of PLA. The synergistic effect of nanofillers and plasticizer achieved the balance between stiffness and toughness with well-controlled crystallization.

## 1. Introduction

Poly (lactic acid) has been the subject of much attention because of its outstanding performance in terms of high stiffness and strength, biodegradability and thermal processability and is often seen as an alternative to traditional petrochemical plastic [1,2]. However, PLA products, under practical processing conditions, are well known to show low crystallinity or are in an amorphous form due to the intrinsic slow crystallization rate, which limits wider applications in sectors, such as automotive and packaging fields [3]. By and large, commercial PLA products with a high molar mass are usually produced from lactide, the ring-formed dimer of lactic acid, via the ring-opening polymerization (ROP) route [4]. As an important intermediate for the industrial production of PLA, lactide exists in three different forms, l,l-lactide, d,d-lactide and l,d-lactide, due to the chiral nature of lactic acid. Commercial PLA is made of copolymers of poly(l-lactide) (PLLA) and poly(d,l-lactic acid) (PDLLA). The l-isomer constitutes the main fraction of PLA derived from renewable sources, since the majority of lactic acid from biological sources exists in this form. Gupta and Kumar [5] reviewed various aspects of PLA synthesis and mentioned the kinds of catalysts in the production of PLA. Specific catalysts can lead to heterotactic PLA, which has been found to show crystallinity. It has been reported [6] that PLA can crystallize in three forms (α, β and γ), depending on the composition of the optically-active l- and d,l-lactide, and the α phase is more stable and has a melting temperature of 180 °C compared to the β phase, with 175 °C [7].

The degree of crystallinity, which has profound effects on the structural, thermal, barrier and mechanical properties, depends on the ratio of d to l enantiomers used. PLA with l-form content greater than 90% tends to crystalline, while those with lower optical purity are amorphous. Furthermore, a suitable selection of the PLA commercial grade with different l-/d- ratios is critical for the requirement of the conversion processing conditions and specific properties.

In processes, such as injection molding, where the orientation is limited with a high cooling speed, it is much more difficult to develop significant crystallinity, and thus, formulation or processing modifications are necessary. Alternatively, nucleating agents can be added to PLA for the promotion of crystallinity via traditional processing, such as injection molding, under a suitable thermal history and cycle time. Basically, the normal nucleating agents reported by some groups are various kinds of inorganic nanoparticles, such as talc, sodium stearate, calcium lactate [8], montmorillonite (MMT) and carbon nanotubes (CNT) [9,10]. It is shown that the crystallization half-time can be required by more than one order of magnitude to less be than 1 min when 1% talc is added [8]. Another reported potential nucleating agent in the literature is the stereocomplex of PLLA and Poly(d-lactic acid) (PDLA). Yamane and coworkers [11] analyzed the crystallization behavior of PLLA with PDLA as a nucleating agent, which formed a large stereocomplex crystalline and effectively increased the number of PLLA spherulites and then the overall crystallization rate. To sum up, the crystallization behavior [12] of PLA depends on the component isomer, processing temperature, annealing time and molecular weight. In general, crystallinity control of injection-molded PLA can be achieved by optimization of the processing parameters (thermal history) and the formulation of materials (stereochemistry). A better understanding of the crystallization behavior and its effects on the mechanical properties is critical for PLA to extend its application.

However, regardless of the component isomers, both the amorphous and crystalline polylactides show brittle behavior at room and body temperature during application as films, fiber or biomedical materials [13]. The toughness improvement is a crucial necessity for many consumer applications, such as food package. Numerous approaches, such as plasticization, block copolymerization, blending with tough polymers and rubbers, have been adopted to improve the toughness of brittle PLA bioplastic. Therefore, plasticization of PLA composites with various kinds of low molecular weight compounds to optimize the mechanical properties is an important field of research. For example, polyethylene glycol (PEG), polypropylene glycol (PPG), glycerol and citrate ester are investigated as plasticizers for PLA to lower its glass transition temperature, increase ductility and improve processability [14,15,16]. However, the major drawbacks of those modifications are the consequent decreases in the strength and modulus of the toughened PLA. There is another limitation for wider PLA industrial applications, which is its poor thermal resistance and limited gas barrier properties, especially in the packaging field. Therefore, preparing a PLA-based material having a good stiffness-toughness balance with high bio-based PLA content, which keeps its original biocompatibility and biodegradability, is one of the big challenges.

In this work, the combination of plasticizer and nucleating agents with PLA aims at the synergistic effects of enhanced crystallinity and reduced injection molding cycle time for PLA products. The crystallization behavior of PLA will be investigated by both DSC non-isothermal and isothermal analyses. The nucleating effect of different nanofillers is discussed according to crystallization parameters, such as the half-crystallization time, crystallinity and the cold crystallization temperature of PLA composites. The relationship between crystallinity and mechanical properties was also investigated by dynamic mechanical thermal analysis (DMTA) and tensile measurements.

## 2. Results and Discussion

### 2.1. Isothermal Crystallization Study

**Figure 1:**
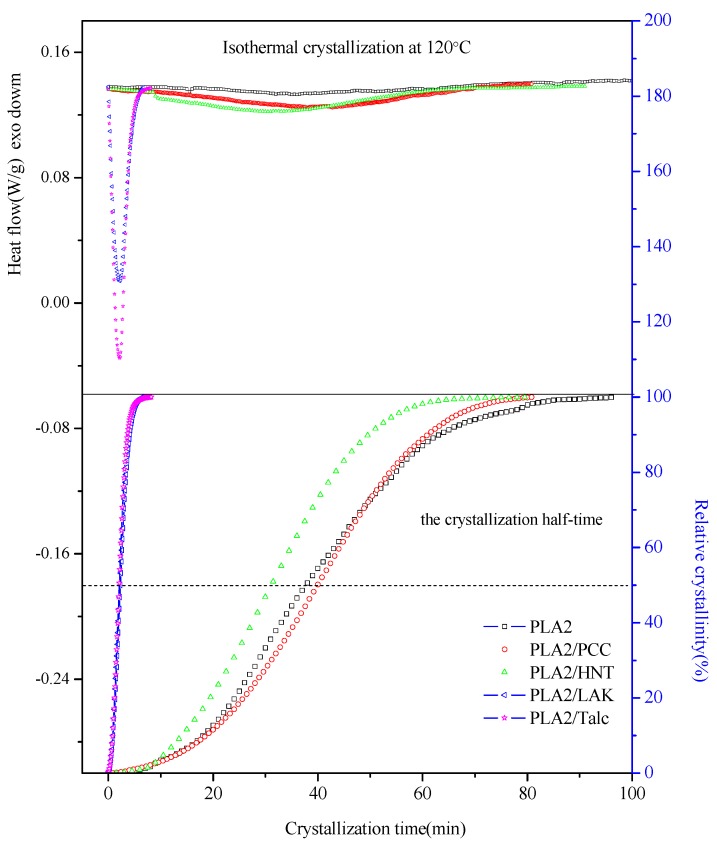
Isothermal crystallization study of PLA2 binary composites with different fillers. (PCC: precipitated calcium carbonate; HNT:halloysite nanotubes; LAK: sulfates).

Firstly, PLA2 nanocomposites with different fillers were prepared to compare the nucleating effect on the isothermal crystallization behavior of PLA2. In the literature [17], the crystallization temperatures have been investigated, and PLA crystallized in the temperature region from 80 °C to 120 °C has been reported. In this work, the isothermal crystallization temperature was chosen to be 120 °C, and the DSC tests of selected PLA2 composites were analyzed. PLA2 shows a wide peak with the longest crystallization time, as expected in Figure 1, while the PLA2/LAK (sulfates) and PLA2/talc systems have sharper isothermal crystallization peaks in the DSC traces.

Furthermore, the isothermal crystallization behaviors of PLA2 and PLA2 composites were further studied according to the Avrami equation, as follows:

(1) $$1-X_{t}=\exp(-kt^{n})$$

where *X_t_* is the relative degree of crystallinity at certain crystallization time *t*, *n* is the Avrami exponent depending on the nature of nucleation and the growth geometry of the crystals and *k* is the crystallization rate constant involving both the nucleation and growth rate parameters. Figure 1 is the relative crystallinity of PLA2 composites *versus* the crystallization time. The half-crystallization time *t*_0.5_ is defined as the time with 50% relative crystallinity, as marked in Figure 1. The values of the Avrami exponent *n* and the crystallization rate *k* can be derived from Figure 1 based on the Avrami equation and the curves of log[−ln(1 − *X_t_*)] against log(*t*), which remains in good linear regression lines, and are shown in Supplementary Information Figure S1. The crystallization parameters for PLA2 composites are shown in Table 1. The crystallization process at 120 °C can approach almost a similar crystallinity of about 30% for all PLA2 composites. However, the crystallization half-time and crystallization rate *k* in Table 1 indicated that the addition of various fillers has different nucleating effects on the isothermal behavior of PLA2. The addition of precipitated calcium carbonate (PCC) and Halloysite natural nanotubes (HNT) has little effect on increasing the crystallization rate and shows similar DSC traces with that of pure PLA2 in Figure 1. Inversely, talc and LAK act as much better nucleating agents. The *t_0.5_* is decreased from 37.5 min of pure PLA2 to about 2.1 min with talc under the same thermal condition. In fact, the addition of only 1 wt % LAK to PLA shows the best increase of the crystallization rate based on the DSC curves. The Avrami exponent *n*, which is the slope of the linear lines in Supplementary Information Figure S1, ranges between two and three in Table 1, which means that there is not much change of the PLA crystallization mechanism, including the crystal growing geometry.

**Table 1:** Comparison of the isothermal parameters of PLA 2002D binary composites with different fillers.

Sample Code	∆Hc (J/g)	Xc (%)	n	log(*k*)	*t_0.5_* (min)
PLA2002D	/	/	2.45	−4.03	**38**
PLA2/PCC	29.14	31.3	2.56	−4.23	**40**
PLA2/HNT	28.58	30.7	2.44	−3.79	**31**
PLA2/talc	29.50	31.7	2.05	−0.81	**2.1**
PLA2/LAK	25.43	27.3	2.04	−0.89	**2.2**

Notes*:* symbol “/” means no value for ∆Hc and Xc.

### 2.2. Non-Isothermal Crystallization Study

**Figure 2:**
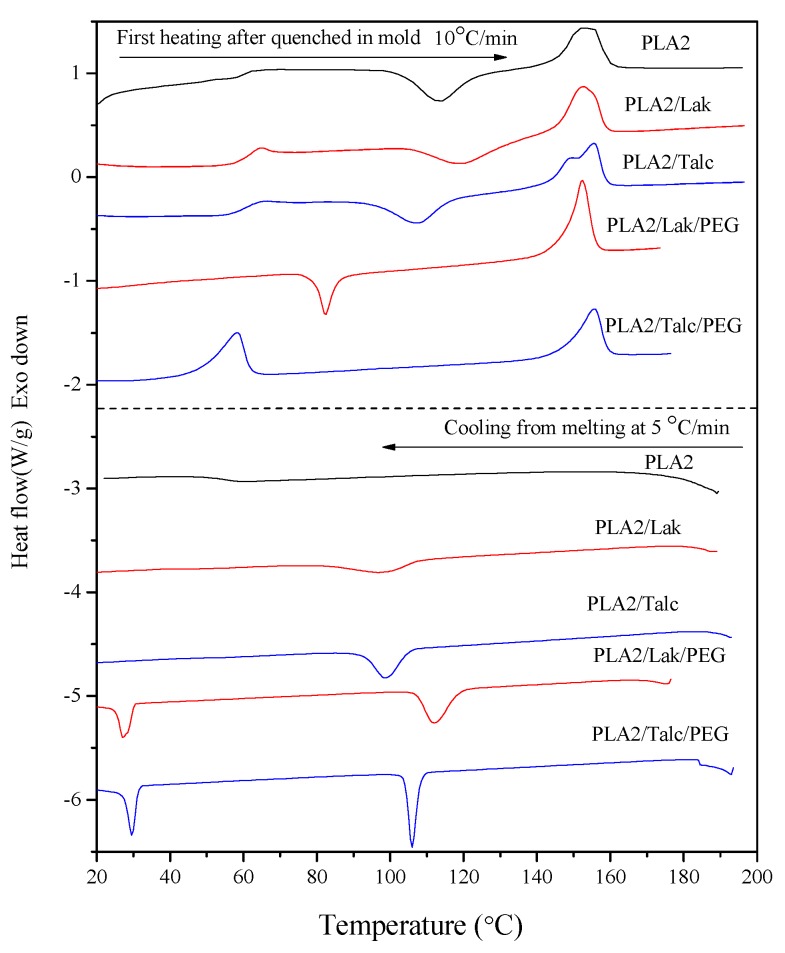
DSC traces of the heating-cooling cycle for PLA molded composites. (Exo means exothermal direction).

Furthermore, the non-isothermal DSC cold crystallization behavior was also studied for PLA2 composites with the two good nucleating agents: LAK and talc. The DSC traces of pure PLA2 and PLA2 composites after being quenched at a certain temperature from the molten state are shown in Figure 2. It is obvious that both pure PLA2 and PLA2 composites have the typical thermal transition peaks during the heating process: the glass transition temperature peak (T_g_), cold crystallization peak (T_cc_) and the two overlapped melting peaks (T_m1_ and T_m2_). The double melting points are also recorded in the literature [18], due to the slow rate of crystallization and the reorganization of crystalline PLA. The main difference in the DSC curve of PLA2 composites with that of pure PLA2 is the lower T_cc_. This can be explained by the fact that the addition of nucleating agents initiates the crystallization at a lower T_cc_ and also produces more perfect crystalline PLA2. To determine the crystallinity levels by DSC, the value that refers most to the melt enthalpy at 100 percent is 93 J/g [19]. 

In practical processing, due to the relatively high T_g_ of PLA, how to decrease the mold temperature and the cycle time is a big challenge. In general, the plasticization of PLA, which leads to lower T_g_, is applied in practical processing technology to solve this problem. Therefore, the addition of the plasticizer PEG to PLA composites was investigated. The DSC traces of PLA ternary composites with 20% PEG in Figure 2 show a much smaller cold crystallization peak or even no cold crystallization peak (of PLA/PEG/talc) during the first heating process, which indicated that the PLA matrix has already crystallized to a certain degree via the quenching process from the molten state in the mold (60 °C for 2 min). The crystallinity of those PLA2 composites is recorded in Supplementary Information Table S2 based on the DSC traces together with other T_g_, T_c_, T_m_ and the enthalpies of each peak. The higher crystallinity of PLA will be desirable for injection molded articles, which have good thermal stability and can offset the drawbacks of plasticized PLA composites with lower stiffness and strength. The thermal resistance analysis will be discussed in the following DMTA results.

The cooling process of PLA2 composites from molten states is also investigated by DSC tests in Figure 2. Pure PLA2 shows no peak under such a low cooling speed and is amorphous, as expected, while a wide small peak appears for PLA2 binary composites with only a filler, especially for talc. Additionally, with the plasticizer PEG in the PLA2 binary composites, there is the appearance of sharp melt crystallization peaks at a higher temperature. The enthalpy of this melt crystallization peak is recorded in Supplementary Table S2, and the crystallinity can reach the maximum as reported in the literature [20].

We also recorded the second heating process of PLA composites in Supplementary Table S2. PLA/talc shows the best enhanced crystallization behavior without the cold crystallization peak. In other words, the PLA/talc binary sample approached full crystallization during the cooling process with a speed of 5 °C/min, while PLA/LAK only achieved partial crystallization of PLA. The DSC traces of PLA ternary composites with the plasticizer PEG show no cold crystallization peak during the second heating, which showed the enhanced crystallization of nucleating agents and the chain mobility of PLA2 due to the plasticizer. The crystallization behavior of PLA is promoted by the addition of nucleating agents, and also, the processing technology is simplified by the addition of the plasticizer, which means that the molding temperature is lower and the cycle time is shorted. Another possible question is the influence of the plasticizer on the mechanical properties and heat resistance of PLA composites, which will be investigated in the following sections.

**Figure 3:**
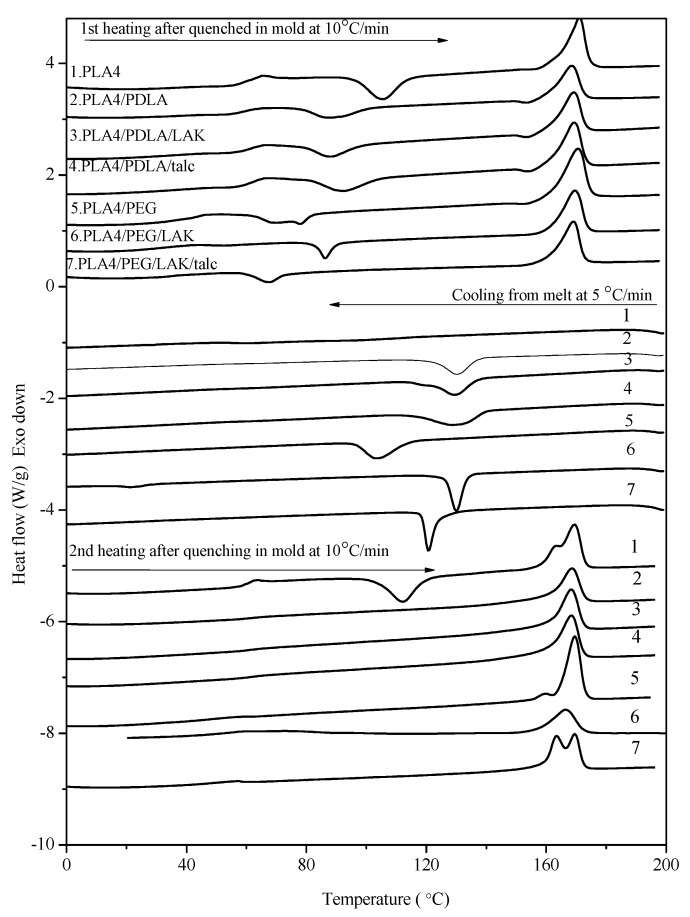
DSC traces (heating-cooling-reheating) of PLA 4032D composites.

PLA4 has a different thermal behavior compared to that of PLA2, as shown in Figure 3, due to the lower d-lactide content in the polymer. The glass transition temperature is the same, 64.8 °C, while PLA4 has a much higher melting point of 170 °C. Following the same preparation process, PLA4 can achieve a crystallinity of about 9%, while this is only 4% for PLA2, as shown in Table S2. In the literature, PDLA/PLLA stereocomplexes are reported as very efficient nucleating agents for PLLA, with increases in both the crystallization rate and the crystallinity, the latter up to 60 percent [21]. Self-nucleation is considered to be an ideal case for homopolymer crystallization due to an optimum dispersion of crystallites and the favorable interactions between the polymer melt and the polymer crystal fragments. In this work, the nucleating effect of PDLA is compared with that of LAK and talc, concluded above to be an efficient nucleating agent, and the non-isothermal DSC traces of PLA4 are shown in Figure 3. PLA4 composite prepared with 10% PDLA shows a similar DSC thermal trace as that of PLA/LAK or PLA/talc. During the first heating process, the cold crystallization peak of all of those PLA/agent binary composites is about 87 °C, almost 20 °C lower than that of pure PLA4. The crystallization behavior of PLA4 from melting is also promoted by the addition of PDLA, LAK and talc, with the melt crystallization peak at about 130 °C, the enthalpy of which approaches the maximum for PLA4. Different from talc and LAK, PDLA acts as a nucleating agent because of the formation of the PLA stereocomplex. This was confirmed by DSC tests in the Supplementary Information Figure S2. The melting peak of the PLA stereocomplex is about 225 °C, which is about 50 °C higher than that of PLA4 and 40 °C higher than that of PDLA. The formed stereocomplex acts as heterogeneous sites for the facilitated PLA crystallization behavior. The enhance crystallinity can also be revealed by the second heating process of PLA composites, which shows no cold crystallization peak in all of the prepared PLA composites. Again, this non-isothermal DSC study confirms that PLA4 itself has a faster crystallization rate than PLA2 under the same quenching condition. We can also conclude that talc and LAK show an effective promotion of PLA crystallization behavior due to the heterogeneous nucleating agents, while the addition of PDLA to PLA results in the formation of the PLA stereocomplex acting as another type of nucleating agent.

Furthermore, the addition of the plasticizer PEG to PLA4 with nucleating agents is also investigated by DSC in Figure 3. The T_cc_ peak of pure PLA in the first heating is around 110 °C, while PLA4 composites with both nucleating agents and PEG have T_cc_ all lower than 80 °C. The second heating process of PLA4 composites shows no cold crystallization peak any longer, due to the presence of nucleating agents and the plasticizer. However, two melting peaks appear at 163 and 170 °C in the second heating of both pure PLA4 and PLA4/PEG/LAK. These two melting peaks indicated that PLA4 with PEG crystallized from melting is not stable, and two different crystals formed during cooling at 5 °C/min. The differences of the second heating process between PLA2 and PLA4 composites in Figure 2 and Figure 3 present that PLA4 has better crystallization in the cases of being by itself and also with fillers.

### 2.3. DMTA Tests

**Figure 4:**
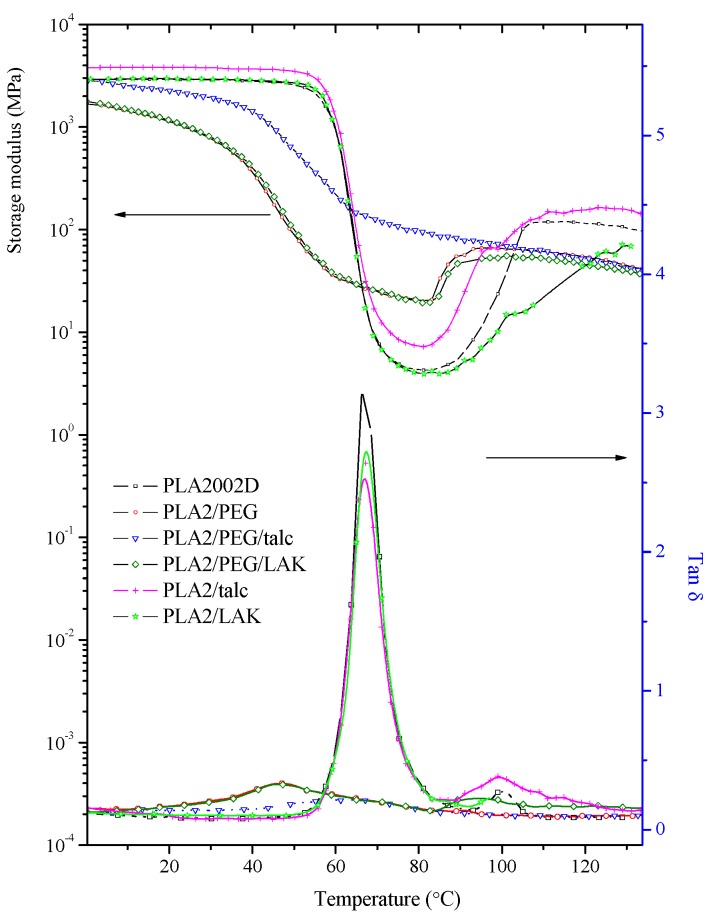
Temperature dependence of the storage modulus and Tanδ of selected PLA2 composites.

The variation of the storage modulus and tanδ of PLA2 and its composites as a function of temperature is illustrated in Figure 4. Basically, the storage modulus of PLA composites relates to the dynamic mechanical properties of PLA at different temperatures, depending on the interface adhesion between PLA and fillers, the loading of inorganic fillers and the crystallinity of the PLA matrix. Pure PLA has the typical curve of the storage modulus: a plateau firstly until the glass transition temperature, accompanied by a sharp decrease, then an increase again, due to the cold crystallization. The first drop corresponds to the main tanδ peak, defined as T_g_, which is different from that determined by the DSC technique, and the other one related to the increase of the storage modulus at high temperatures is the cold crystallization temperature. At room temperature, the addition of different fillers to PLA2 results in a similar storage modulus as that of pure PLA2. At high temperatures above T_g_, PLA/talc has a relatively higher storage modulus than that of neat PLA, which is attributed to the high stiffness and strength of talc and also the increased crystallinity of PLA/talc. Therefore, the combination of talc reinforcement and the enhanced crystallization of the PLA matrix will lead to the better mechanical properties of this PLA binary composite.

**Figure 5:**
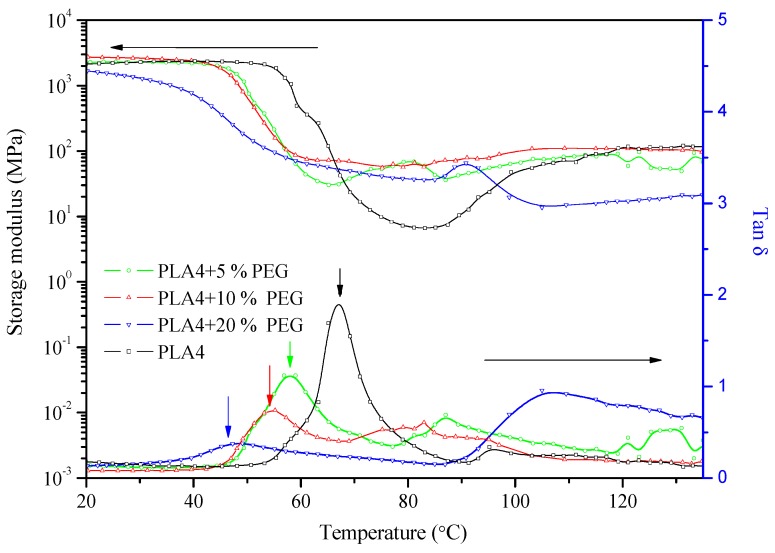
Temperature dependence of the storage modulus and tanδ of PLA4 binary composites with PEG.

However, with the plasticizer PEG in PLA2, the storage modulus initiates a decrease from a lower temperature, and this is reflected by the decrease of the damping peak. Pure PLA2 represents a glass transition temperature of 67 °C with a sharp decrease of the storage modulus. The addition of only fillers to PLA has no effect on the glass transition temperature, which indicated the stability of PLA chain mobility. Some researcher [22] reported a higher T_g_ of PLA binary composites, explained by the strong interfacial adhesion between the filler and PLA chain, which restricted the mobility of the PLA matrix. Conversely, the addition of PEG to PLA shows a decreased magnitude of tanδ in comparison to virgin PLA and also the shift of the damping peak to a lower temperature according to the PEG content. Figure 5 is the temperature dependence of the storage modulus and tanδ of PLA4/PEG binary composites with different PEG contents. It is obvious that the addition of PEG decreases the glass transition temperature from 67.3 °C to 57.5, 53.2 and 47.3 °C with 5 wt %, 10 wt % and 20 wt % PEG in the composites, respectively. Another result is that the PLA/PEG binary composite can effectively eliminate the effect of the cold crystallization of PLA, which indicates the enhancement of the PLA chain mobility and the higher crystallinity of PLA/PEG composites. Those DMTA results confirm the increased crystallinity with the addition of PEG in PLA. According to the temperature dependence of the tanδ peaks of PLA binary composites, there is no phase separation between PLA and PEG, at least with 20 wt % of the plasticizer. The crystallization and phase separation in blends of high stereoregular PLA with PEG have been studied in the literature, and both PLA and PEG crystallized during slow cooling. The proportionality between PEG crystallinity and PLA crystallinity indicated that the PEG that was trapped in the intraspherulitic regions crystallized much more rapidly than PEG in the interspherulitic amorphous regions [13]. The plasticization effect is enhanced by higher plasticizer content and a decrease of the molecular weight. The plasticizers enhanced the segmental mobility of PLA chains, increasing the mobility of the amorphous to the plastic deformation. Therefore, the decreased T_g_ is also assumed to decrease the tensile strength and increase the elongation-at-break, which will be discussed in the mechanical properties section.

**Figure 6:**
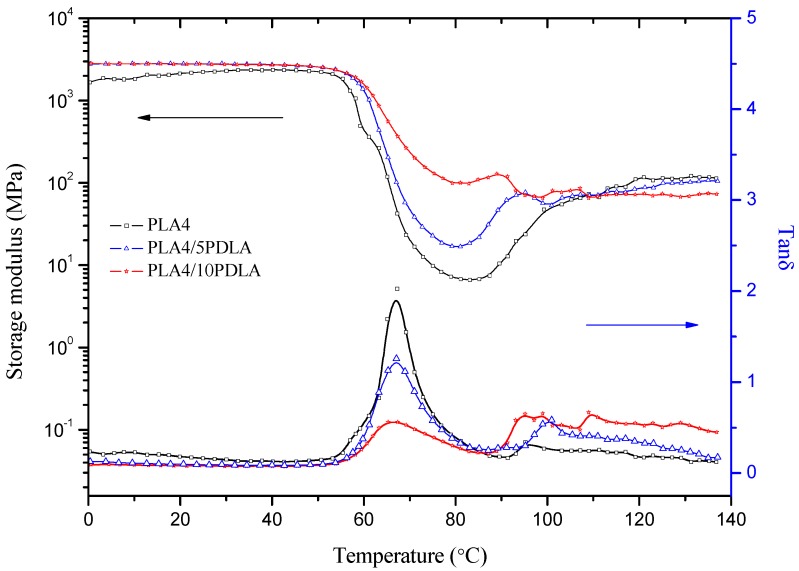
Temperature dependence of the storage modulus and tanδ of PLA4 binary composites with Poly(d-lactic acid) (PDLA).

As discussed above, PDLA is applied in this work as another effective nucleating agent. PLA4 binary composites with only PDLA are also studied by DMTA measurements. Figure 6 shows the storage modulus and tanδ of PLA4/PDLA as a function of temperature. With the increase of PDLA content in the PLA4, the storage modulus curve above T_g_ shows no cold crystallization effect, which means a higher crystallinity of PLA/10PDLA due to the nucleating influence of PDLA. The damping peak temperature stays the same in PLA/PDLA composites compared with that of pure PLA. 

The combination of nucleating agents and plasticizers with PLA2 shows different thermal-dynamic behaviors. In Figure 4, PLA2/PEG/filler ternary composites have similar storage modulus curves, which keep decreasing as the temperature increases. The content of PEG remains the same and is 20% in those ternary composites. PLA/PEG/talc has a relatively higher storage modulus at the same temperature compared to that of PLA/PEG/LAK, which is mainly attributed to the high filler content of talc at 10%. In the case of the PLA/PEG/talc ternary composite, the storage modulus keeps decreasing, due to the lower temperature without cold crystallization at a higher temperature. In other words, the DMTA measurement matches the DSC tests well regarding the nucleating effect of talc and the faster crystallization behavior in the presence of both nucleating agent and plasticizer. At least under the mold temperature of 60 °C, PLA can reach a relatively higher crystallinity via a short cycle time during the molding. This result is quite important for the PLA composites with high mechanical properties and thermal resistance. 

### 2.4. Mechanical Properties

The tensile performance of all of the PLA2 composites prepared is reported in the Table 2. The average tensile strength, Young’s modulus and elongation-at-break of neat PLA2 are about 65.15 MPa, 3.48 GPa and 3.29%, respectively. PLA4 has a relatively higher Young’s modulus and tensile strength than PLA2, as shown in Table 2, which matches well with the thermal behavior of both types of PLA. The Young’s modulus of PLA2 binary composites with fillers increased due to the inherent high stiffness strength of the fillers, while the elongation-at-break of those samples remains at the same level as that of pure PLA, around 3%. However, some samples show a little bit of an increase of the elongation-at-break, from 3.2% to about 5%, which can be attributed to the toughening effect of the well-dispersed fillers in the polymer matrix. Toughened plastics with nanofillers have been reported [23], and the explanation is that a suitable interfacial adhesion between fillers and polymer produces easy debonding and, further, the formation of microvoids for the adsorption of break energy. The weaker interfacial adhesion between fillers and PLA polymer explains the lower tensile strength of PLA/filler binary composites.

**Table 2:** Mechanical properties of PLA composites.

PLA Sample Code	Composition	Young’s Modulus (GPa)	Tensile Strength (MPa)	Elongation at Break (%)
PLA2	100	3.48	65.15	3.29
PLA2/LAK	99/1	3.98	64.37	5.30
PLA2/talc	95/5	/	59.87	2.04
PLA2/PEG	80/20	/	29.59	202.7
PLA2/PEG/LAK	79/20/1	1.42	29.44	177.0
PLA2/PEG/talc	75/20/5	/	35.64	132.6
PLA4	100	3.83	69.06	2.48
PLA4/PDLA	99/1	3.65	71.57	4.30
PLA4/PDLA	95/5	3.61	67.40	2.98
PLA4/PDLA	90/10	4.19	68.79	3.32
PLA4/PEG	95/5	3.55	58.45	2.83
PLA4/PEG	90/10	3.16	53.83	10.25
PLA4/PEG	80/20	1.12	20.08	174.0
PLA4/PDLA/LAK	94/5/1	3.18	64.45	5.34
PLA4/PDLA/talc	90/5/5	3.86	64.22	2.28
PLA/PEG/PDLA	79/20/1	1.08	30.40	210.30
PLA/PEG/LAK	79/20/1	1.02	31.90	195.20
PLA/PEG/LAK/talc	79/10/1/10	4.24	46.10	174.88

Notes: Symbol “/” means no value available.

Pure PLA, as a semicrystalline polymer, is quite brittle, and the fracture mechanism is mainly due to the craze fracture of PLA. The plasticized PLA with increased PLA chain mobility obviously decreases the stress required for craze initiation and growth, which resulted in a lower tensile strength and a higher elongation-at-break for the mechanical properties.

The addition of PEG in PLA polymer shows an impressive increase of the elongation-at-break. The mechanical properties of PLA4 with different PEG contents indicate that the elongation-at-break of PLA4/PEG increased with the plasticizer content. The PLA4/PEG5 and PLA4/PEG10 exhibited a rather small elongation-at-break of 2.83% and 10.25%, respectively. As the PEG content increases to 20%, the break deformation is about 174%. The toughness improvement of PLA4 with PEG is obvious, while the tensile strength of PLA4/PEG decreases with the increasing content of PEG, which is about 20.08 MPa with 20% plasticizer. In order to balance the toughness and strength of PLA nanocomposites, both the nanofillers and plasticizers were added together in the PLA matrix. Table 2 and Figure S4 show the mechanical properties of the PLA/PEG/nanofillers, and the best mechanical performance of those is that of PLA/PEG/talc/LAK. The tensile strength of PLA/PEG/talc/LAK is about 46.10 MPa with an elongation-at-break of 174.88%. In this composition, PEG acts as a plasticizer to decrease the glass transition temperature and improve the chain mobility, while LAK acts as a nucleating agent to increase the crystallinity. Talc works as a reinforcement nanofiller to maintain the high modulus and stiffness of pure PLA. The mechanical results also confirmed the enhanced crystallinity characterized by DSC and DMTA.

## 3. Experimental Section

### 3.1. Materials

Extrusion grade PLA 2002D (4% d-lactide, 96% l-lactide content, nominal average molecular weight Mw = 199,590, MFR (melt flow rate) = 5–7 g/10 min) and PLA 4032D (1.4% d-Lactide, 98.6% l-Lactide content, nominal average molecular weight Mw = 200,000, MFR 5–7 g/10 min) was purchased from Natureworks^®^ (Natureworks LLC, Minnetonka, MN, USA) in pellet form and marked as PLA2 and PLA4 in the following text. Precipitated calcium carbonate (PCC) coated with 13.5 wt % stearic acid [24] was supplied with an average size of 70 nm by Solvay (Solvay Advanced Functional Minerals, Salin de Giraud, France). Halloysite natural nanotubes were supplied by Imerys Tableware Ltd. (Auckland, New Zealand). LAK 301, a type of aromatic sulfonate derivative, was kindly supplied by Takemoto Oil & Fat Co., Ltd, Gamagori, Japan. Micro-sized talc, coded Jetfine^®^1A, was supplied by Luzenac (Imerys, Paris, France). PDLA was chosen as the nucleating agent in this work. Polyethylene glycol (PEG) with a nominal molecular weight of 6000 g/mol, used as a plasticizer, was purchased from Sigma-Aldrich (Shanghai, China).

### 3.2. Sample Preparation

The PLA binary or ternary composites studied in this work were prepared by melt extrusion with the MiniLab II Haake Rheomex CTW 5 conical twin-screw extruder (Thermo Scientific Haake GmbH, Karlsruhe, Germany), at a screw rate of 90 rpm/min, a cycle time of 30 seconds and with the extrusion barrel working at a temperature of 190 °C and 210 °C for PLA2 and PLA4, respectively. Prior to the extrusion, the PLA and fillers were dried at 80 °C in a vacuum oven for eight hours. The compositions of PLA binary and ternary composites with nucleating agents and plasticizer are listed in Supplementary Information Table S1.

After extrusion, the molten materials were transferred through a preheated cylinder to the Haake MiniJet II mini injection molder (Thermo Scientific Haake GmbH, Karlsruhe, Germany), to obtain ASTM D638 V dog-bone tensile bars used for measurements and analysis. The molding parameters were: barrel, 190 °C; mold, 60 °C; injection pressure, 800 bar; cycle time, 30–120 s.

### 3.3. Characterization

#### 3.3.1. Thermal Properties

The crystallization behavior of PLA composites was investigated by the DSC technique with a TA Q200 instrument (TA Instruments, New Castle, DE, USA) with nitrogen as the purge gas. The non-isothermal program was carried out firstly from 20 °C to 190 °C by a heating rate of 5 °C/min. The sample was kept at 190 °C for 5 min to remove the thermal history and then cooled to room temperature for PLA composites. The sample for the isothermal study of PLA nanocomposite was quenched from 200 °C to the isothermal temperature of 120 °C as the crystallization temperature.

#### 3.3.2. Mechanical Properties

Tensile tests were performed at room temperature, at a crosshead speed of 10 mm/min by an Instron 4302 universal testing machine (Canton, MA, USA) equipped with a 10-kN load cell and interfaced with a computer running the Testworks 4.0 software (MTS Systems Corporation, Eden Prairie, MN, USA, 2011). At least five specimens were tested according to the ASTM D 638.

#### 3.3.3. Dynamic Mechanical Properties

Dynamic mechanical thermal analysis was carried out on pure PLA and PLA composites by means of a Gabo Eplexor^®^ 100N (Gabo Qualimeter GmbH, Ahlden, Germany). Test bars were cut from the tensile bar specimens (size: 20 × 5 × 1.5 mm) and mounted in tensile geometry. The dynamic storage and loss modulus were recorded with a constant frequency of 1.00 Hz as a function of temperature from −100 °C to 150 °C with a heating rate of 2 °C/min.

## 4. Conclusions

PLA nanocomposites were prepared by melt extrusion processing technology. The effect of heterogeneous nucleation was accessed by adding CaCO3, HNT, LAK and talc as potential agents. All of those nanofillers acted as nucleating agents for PLA, as confirmed by the DSC measurements, while LAK and talc showed much better effects on the enhanced PLA crystallization behavior. The enhanced crystallization under the traditional molding process of PLA nanocomposites was attributed to the effective nucleating agents. PDLA was also compared with other nanofillers as nucleating agents due to the formation of the stereocomplex, and the DSC study indicated that PDLA acted as an effective agent to improve the PLA crystallization behaviors. Plasticized PLA showed that the glass transition temperature decreased with the increase of the PEG content. The mechanical performance of PLA nanocomposites was discussed for both the binary and ternary composites. Nanofillers acted as the reinforcement fillers, while the plasticizer was effective at enhancing the PLA chain mobility. The balance of the stiffness and toughness of PLA nanocomposites was achieved by the combination of both nanofillers and plasticizer. In fact, LAK is a better nucleating agent, while talc is a better reinforcement filler than the other nanofillers.

## Supplementary Materials

Supplementary materials can be accessed at: http://www.mdpi.com/1420-3049/20/01/1579/s1.
