# Supplementary material for: Synergistic Effects of Nucleating Agents and Plasticizers on the Crystallization Behavior of Poly(lactic acid)
Source: Molecules. 2015 Jan 19;20(1):1579–93. doi: 10.3390/molecules20011579 (PMC6272527; doi:10.3390/molecules20011579)
Supplement: Supplementary file 1 [file molecules-20-01579-s001.pdf]

Supplementary Materials

Table S1. Composition of PLA composites in this work.

Sample Code	Polymer	Fillers	Plasticizer
PLA2	PLA2 (100)	0	0
PLA4	PLA4 (100)	0	0
PLA2/PCC	PLA2 (95)	PCC (5)	0
PLA2/HNT	PLA2 (95)	HNT (5)	0
PLA2/talc	PLA2 (95)	talc (5)	0
PLA2/LAK	PLA2 (99)	LAK (1)	0
PLA2/PEG/LAK	PLA2 (79)	LAK (1)	PEG (20)
PLA2/PEG/talc	PLA2 (75)	talc (5)	PEG (20)
PLA4/5PDLA	PLA4 (95)	PDLA (5)	0
PLA4/10PDLA	PLA4 (90)	PDLA (10)	0
PLA4/PDLA/LAK	PLA:PDLA = 95:5	LAK (1)	0
PLA4/PDLA/talc	PLA:PDLA = 95:5	talc (5)	0
PLA4/5PEG	PLA (95)	0	PEG (5)
PLA4/10PEG	PLA(90)	0	PEG (10)
PLA4/20PEG	PLA (80)	0	PEG (20)
PLA4/PEG/LAK	PLA4 (79)	LAK (1)	PEG (20)
PLA4/PEG/LAK/talc	PLA4 (79)	LAK (1) talc (10)	PEG (10)

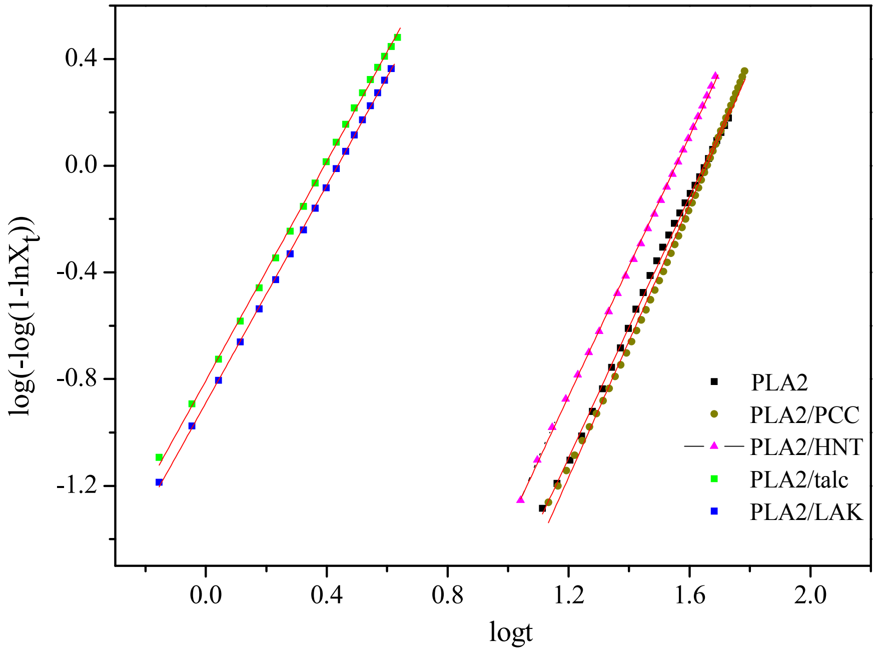

Figure S1. Linear Avrami plots of the selected PLA composites.

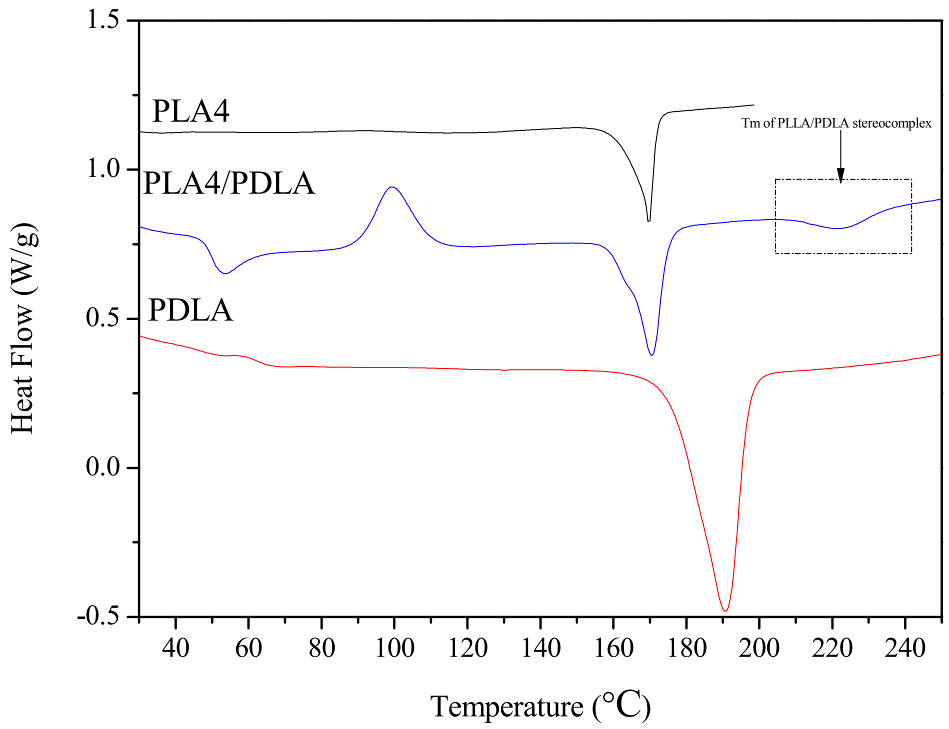

Figure S2. DSC traces of PLA, PDLA and their blend.

Table S2. Crystallization parameters of PLA composites.

Sample	1st Heating (10 °C/min)						Cooling (5 °C/min)		2nd Heating (10 °C/min)					
	T_g	T_{cc}	T_m	ΔH_{cc}	ΔH_m	X_c (%)	T_{cm}	ΔH_{cm}	T_g	T_{cc}	T_{m1}	T_{m2}	ΔH_{cc}	ΔH_m
PLA2D	64.8	113.9	151.4/155.4	22.27	24.56	2.46	/	/	No test					
PLA4D	64.8	105.7	171.1	31.79	40.17	9.01	/	/	62.9	112.2	163.3	169.5	30.55	41.14
PLA2/LAK	65.4	120.7	153	19.05	28.73	10.50	107.3	22.36	62.6	110	150.7	156.2	3.37	29.03
PLA2/talc	65.8	101	156	19.35	28.67	11.12	104.8	27.03	61.8	/	149.5	156.5	/	29.13
PLA2/PEG/LAK	/	86.2	156.0	14.92	28.87	18.97	115.1	28.37	/	/	149	156	/	29.53
PLA2/PEG/talc	/	no	155.5	0	13.05	20.02	111.9	24.38	/	/	147.1	155.8	/	26.25
PLA4/PDLA	64.8	88.4	168.5	18.42	31.82	15.99	130.2	/	68.4	/	/	168.6	/	42.8
PLA4/LAK	66.6	102.9	170.5	24.66	37.50	13.93	128.3	42.2	/	/	/	167.8	/	49.18
PLA4/PDLA/LAK	66.1	92.8	169.3	19.93	40.06	23.00	129.5	44.17	64.5	/	/	168.4	/	44.85
PLA4/PDLA/talc	65.8	88.6	169.2	17.36	37.26	23.75	129.5	39.42	65.9	/	/	169.19	/	40.46
PLA4/PEG	/	77.9	170.5	16.35	49.78	44.88	103.5	39.00	/	/	159.3	169.5	/	44.57
PLA4/LAK/PEG/Talc	/	67.9	169.1	9.17	36.61	37.31	120.8	36.9	/	/	163.3	169.4	/	39.33

T_g—glass transition temperature; T_{cc}—cold crystallization temperature; T_m—melting temperature; ΔH_{cc}—cold crystallization enthalpy; ΔH_m—melting enthalpy; X_c—crystallinity; T_{cm}—melting crystallization temperature; ΔH_{cm}—melting crystallization enthalpy; \ means no detectable value.

Table S3. Mechanical properties of PLA composites.

PLA Sample Code	Composition	Young's Modulus (GPa)	Tensile Strength (MPa)	Elongation at Break (%)
PLA2	100	3.48	65.15	3.29
PLA2/LAK	99/1	3.98	64.37	5.30
PLA2/talc	95/5	/	59.87	2.04
PLA2/PEG	80/20	/	29.59	202.7
PLA2/PEG/LAK	79/20/1	1.42	29.44	177.0
PLA2/PEG/talc	75/20/5	/	35.64	132.6
PLA4	100	3.83	69.06	2.48
PLA4/PDLA	99/1	3.65	71.57	4.30
PLA4/PDLA	95/5	3.61	67.40	2.98
PLA4/PDLA	90/10	4.19	68.79	3.32
PLA4/PEG	95/5	3.55	58.45	2.83
PLA4/PEG	90/10	3.16	53.83	10.25
PLA4/PEG	80/20	1.12	20.08	174.0
PLA4/PDLA/LAK	94/5/1	3.18	64.45	5.34
PLA4/PDLA/talc	90/5/5	3.86	64.22	2.28
PLA/PEG/PDLA	79/20/1	1.08	30.40	210.30
PLA/PEG/LAK	79/20/1	1.02	31.90	195.20
PLA/PEG/LAK/talc	79/10/1/10	4.24	46.10	174.88

Notes: Symbol "/" means no value available.
